# Effects of oregano (*Lippia origanoides*) essential oil supplementation on the performance, egg quality, and intestinal morphometry of Isa Brown laying hens

**DOI:** 10.14202/vetworld.2021.595-602

**Published:** 2021-03-09

**Authors:** Swanny Y. Ramirez, Lina M. Peñuela-Sierra, Maria A. Ospina

**Affiliations:** 1 Department of Veterinary Medicine and Animal Science , Faculty of Veterinary Medicine and Animal Science, University of Tolima, Ibagué, Colombia; 2 Department of Biology, Faculty Science, University of Tolima, Ibagué, Colombia

**Keywords:** egg quality, growth promoter antibiotic, intestinal morphometry, oregano essential oil, performance

## Abstract

**Background and Aim:**

The use of antibiotics as growth promoters in the feed of poultry, has contributed to an increase in the antimicrobial resistance of foodborne pathogens worldwide. Hence, the development of new effective alternatives to antibiotics that do not hinder productivity is imperative. For this, the aim of the present study was to determine whether oregano essential oil (OEO) extracted from *Lippia origanoides* is a suitable alternative to growth-promoting antibiotics (GPAs) for improving the performance, egg quality, and intestinal morphometry of ISA Brown laying hens.

**Materials and Methods:**

A total of ninety-six 70-week-old ISA Brown laying hens were randomly assigned to four treatment groups with four replicates per treatment and six hens per replicate. The treatments consisted of four different diets that were formulated according to the nutritional requirements of the genetic line and the production phase with and without the addition of GPA and OEO: NC, which did not contain OEO or GPA; GPA, which included 50 ppm zinc bacitracin as a GPA; 80OEO, which included 80 ppm OEO and no GPA; and 150OEO, which included 150 ppm OEO and no GPA.

**Results:**

All of the OEO and GPA treatment groups had a better feed conversion ratio than the NC group. However, the addition of 150 ppm OEO to the diet improved the percentage egg production and egg mass, as well as the external and internal quality of the egg compared with the other treatments. In addition, both the 80OEO and 150OEO treatments improved the yolk color, shell thickness, and shell color, as well as parameters related to the intestinal morphometry compared with the NC group.

**Conclusion:**

The findings of this study indicate that 150 ppm OEO can be used as a substitute for GPA to improve the performance, egg quality, and parameters related to the intestinal morphometry of ISA Brown laying hens.

## Introduction

The application of sub-therapeutic doses of growth-promoting antibiotics (GPAs) in the poultry industry has allowed the development rate and production yield of the birds to be increased while preventing infections [1] by changing their intestinal microbiota, metabolism, and intestinal function [2]. Feeding is a particularly important factor in commercial poultry farming as it represents approximately 70% of the total cost of production, which has led to increased use of GPAs in the poultry industry [3]. However, this has contributed to an increase in the antimicrobial resistance of foodborne pathogens worldwide [4,5], which is not only compromising the future of world health but is also affecting the food security, development, and economy of individual countries. Consequently, international health organizations have called on all nations to coordinate the development of new strategies to fight antimicrobial resistance through promotion of the “one health” approach, which recognizes the link between the health of humans, animals, and ecosystems [6]. In addition to this global issue, the consumption of poultry products, including eggs, is also increasing due to their high nutritional value and low cost [7]. Consequently, the industry is currently in search of alternatives to GPAs that will help the birds to efficiently assimilate the nutrients contained in their diets, allowing them to continue to achieve high productivity at a low cost and to produce a quality product for the consumer without causing resistance to microorganisms or antibiotic residuals in products of animal origin [8,9].

Functional or nutraceutical foods such as prebiotics, probiotics, plant extracts, organic acids, and essential oils have been developed as alternatives to GPAs [10], and essential oils, in particular, have become an integral alternative for use in animal feed [11,12]. Among the essential oils that are used in the poultry industry, those extracted from oregano (Origanum spp.), thyme (Thymus spp.), and garlic (*Allium sativum*) have been shown to have similar effects to GPAs on weight gain and control of pathogenic bacteria in the digestive tract, improving the quality of the final product without posing a risk to consumers [11]. In broiler chickens, these functional effects have been attributed to the contents of the phenols carvacrol and thymol in the presence of other components such as hydrocarbon monoterpenes, γ-terpinene, and ρ-cymene [13]. However, few studies have investigated these effects in commercial layers of the ISA Brown genetic line [13].

The aim of this study was to evaluate the effects of using oregano essential oil (OEO) extracted from *Lippia origanoides* as an alternative to GPAs on the performance, egg quality, and intestinal morphometry of ISA Brown layers.

## Materials and Methods

### Ethical approval

The current experiment was approved by the Animal Ethics Committee of University of Tolima, Colombia. The study incorporated management procedures to preserve the integrity of the animals, according to resolution 8430 of the Ministry of Health of Colombia.

### Study period and location

The study was conducted from June to December 2018. This experiment was carried out at the La Amparo farm for commercial egg production, which is located in the San Antonio neighborhood of the municipality of Ibagué, Tolima Department, Colombia.

### Study design

The birds were housed in an open shed containing 32 standard two-level, metal, pyramid-type cages (40 cm high×50 cm wide×50 cm deep) at a stocking density of three hens per cage.

A total of ninety-six 70-week-old ISA Brown laying hens were randomly assigned to four treatments, with four replicates per treatment and six birds per replicate. The treatments consisted of four different diets that were formulated to meet the nutritional requirements of the genetic line and the production phase with and without the addition of GPA and OEO: NC, which did not include OEO or GPA as a negative control; GPA, which included 50 ppm zinc bacitracin as a GPA and no OEO as a positive control; 80OEO, which included 80 ppm OEO and no GPA; and 150OEO, which included 150 ppm OEO and no GPA. The basal diet that was formulated for all treatments is shown in Table-1.

**Table-1:** Nutritional composition of the basal diet formulated for the treatments.

Ingredient	Quantity %
Yellow corn	44.10
Soybean meal	25.00
Extruded soybean	7.40
Rice flour	7.00
Wheat grain	5.00
Calcium carbonate M4	4.90
Calcium carbonate M30	4.00
Dicalcium phosphate	1.60
Pre-mix Vit-Min	0.15
Common salt	0.29
DL-Methionine	0.20
Sodium sesquicarbonate	0.11
L-Threonine	0.01
Sodium aluminosilicate	0.20
Fenbendazole 4%	0.01
Choline chloride 60%	0.05
Nutrient analysis (calculated)	
Metabolizable energy Kcal/kg	2840
Crude protein (%)	16.9
Fats (%)	7.1
Fiber CR (%)	3.29
Ash (%)	13.24
Lysine dig birds (%)	0.77
Methionine dig birds (%)	0.42
Threonine dig birds (%)	0.54
Calcium (%)	4.1
Available phosphorus (%)	0.36
Cl (%)	0.21
Na (%)	0.17
Electrolytic balance (mEq/Kg)	221.7

Vitamin mineral premix supplemented by Kg of diet=Vitamin A – 10,000 IU; Vitamin D3 – 2500 IU; Vitamin E – 20 mg; Vitamin K3 – 3 mg; thiamine – 2 mg; riboflavin – 5 mg; niacin – 40 mg; pantothenic acid – 12 mg; pyridoxine – 5 mg; folic acid – 0.75 mg; Vitamin B12 – 0.015 mg; biotin – 0.05 mg; cantaxanthin – 1.9 mg; Apo-Ester – 3 mg; copper – 8 mg; iron – 60 mg; iodine – 1 mg; manganese – 70 mg; selenium – 0.25 mg; zinc – 60 mg

The hens were supplied with the diets for a total of 10 weeks (weeks 70-80 of age), the first 2 weeks of which were used as an adaptation period while the following 8 weeks were used for data collection. Feed was offered daily at a rate of 110 g/bird/day in the form of flour, and water was offered *ad libitum* throughout the experimental period. All birds were weighed at the start of the experiment and then at 15-day intervals until the end of the experiment. To assess the morphometry of the small intestine, 16 birds (one per replicate) were slaughtered by cervical dislocation at the end of the experiment, following Kermanshahi *et al*. [14].

### Source of OEO

A powdered product containing 16% essential oil extracted from *L. origanoides* that had guaranteed concentrations of 8% thymol and 4.9% carvacrol per kg of product was used as a source of OEO.

### Performance of the laying hens

Each hen was weighed at the beginning and end of the experiment and the change in body weight was calculated. In addition, the number and weight (g) of eggs produced were recorded daily by weighing each individual egg with a calibrated Scout^®^ Pro SP2001 precision balance (Ohaus Corporation, NJ, USA). The egg mass was then calculated by multiplying the average egg weight by the egg number, and egg production (%) was calculated by dividing the total egg number by the number of laying hens. Feed consumption was also recorded daily, and the feed conversion ratio was calculated by dividing the total feed intake by the total egg mass. Finally, mortality (%) was determined by dividing the number of dead birds by the total number of birds per treatment and multiplying by 100.

### Egg quality

Measurements of the internal and external egg quality were made on five occasions at 2-week intervals using three eggs per replicate or a total of 12 eggs per treatment. On the day of collection, each egg was marked and weighed with a calibrated Scout® Pro SP2001 precision balance(Ohaus Corporation) and then broken on a flat surface to measure the height of the albumin using a 0.01 mm precision micrometer. The Haugh unit (HU) value, which takes into account the logarithmic relationship between the height of the dense albumin and the weight of the egg, was then calculated using the formula UH=100×log(H+7.57−1.7W^0.37^), where H is the height of the albumin in millimeters and W is the weight of the egg in grams [15].

The color of the yolk was determined through visual comparison with a Roche^®^ colorimetric fan (Hoffmann-La Roche, Basel, Switzerland) and assigned a score on a numerical scale from 1 to 15. The yolk was then separated from the white and weighed to determine the percentage of yolk. The shells were dried at room temperature for 48 h and weighed individually to determine the percentage of shell in relation to the weight of the egg. The percentage of white was then calculated as 100-(percentage of yolk+percentage of shell). The shell thickness was determined with a Mitutoyo^®^ digital micrometer (Mitutoyo America Corporation, Illinois, USA) with an accuracy of 0.001 mm, taking the average value of three points on the shell (wide pole, narrow pole, and equatorial zone) as a reference [16].

The shell color and resistance to breakage were determined on three occasions using three eggs per replicate or a total of 12 eggs per treatment. The shell color of each egg was measured at 10 points on the surface using a HunterLab ColorQuest^®^ XE spectrocolorimeter (HunterLab, Virginia, USA) in the CIE L*a*b* color space, where L represents the luminosity (range=0-100, where 0 corresponds to black and 100 to white), a* indicates the contents of red or green (with positive and negative values indicating the red and green ends of the spectrum, respectively), and b* indicates the contents of yellow and blue (with positive and negative values indicating the yellow and blue ends of the spectrum, respectively) [17]. The breaking strength of the shell was determined using a TA.XTPlus texture analyzer (Stable Micro Systems Ltd., Godalming, UK), whereby the eggs were compressed under controlled conditions until the shell cracked or broke and the force required to break the shell was expressed in Newtons [18].

### Intestinal morphometry

At the end of the trial, four randomly selected birds per treatment (one per replicate) were euthanized by cervical dislocation and the entire gastrointestinal tract was removed. Samples of approximately 2 cm were then collected from the following segments of the small intestine: (1) The apex of the duodenum, (2) half way between the point of entry of the bile duct and Meckel’s diverticulum (jejunum), and (3) 10 cm proximal to the ileocecal junction (ileum). The samples were fixed in a neutral buffered formalin solution for 72 h and then cut into 1 mm sections. These sections were then dehydrated in alcohol and embedded in paraffin blocks, following which 5 mm cross-sections were cut with a microtome and stained with hematoxylin-eosin. At least 10 adjacent villi that were in a straight position were evaluated for each bird by measuring the following dimensions: Villus height (from the apex to the base of the villus), crypt depth (from the base of the villus to the muscle of the mucosa), and villus width (half the height of the villus). The villus height:crypt depth ratio was then calculated [19].

### Statistical analysis

A completely randomized design was used for the statistical analysis of the treatments. Egg quality means and intestinal morphometry were performed by analysis of variance using the following model:

Y*_ij_* = µ + T*i* + ε*ij*

Dónde:

Y*_ij_* = Observation *j* in group or treatment *i*

µ = is the general mean.

T*i* = Fixed effect of group or treatment *i*

ε*ij* = Random error with mean 0 and variance σ^2^.

Likewise, a Duncan test was run to compare all the means of the treatments using the InfoStat statistical program version 2019 [20].

## Results

### Performance of the laying hens

The effects of the treatments on laying performance in terms of the body weight change (g), feed intake (g/hen/day), egg production (%), egg weight (g), egg mass (g/hen/day), and feed conversion ratio (g feed/g egg) are shown in Table-2. The 150OEO treatment group had a significantly higher feed intake than all other treatment groups (p<0.05) and a significantly higher percentage egg production than the NC group (p<0.05). However, there were no significant differences in the weight gain of the birds or the average weight of the eggs among the treatments. The OEO and GPA treatment groups had significantly higher feed conversion ratios than the NC group (p<0.05). There was no significant difference in the % mortality observed during the experimental period among the treatments (p>0.05).

**Table-2:** Effect of the inclusion of oregano essential oil on the performance in Isa Brown hens.

Performance	Treatments					

	NC	GPA	80OEO	150OEO	SEM	p-value
Body weight change (g)	108.33	109.17	108.1	109.58	2.31	0.963
Feed intake (g/hen/day)	104.30^a^	104.42^a^	103.69^a^	108.27^b^	1.08	p<0.05
Egg production %	66.80^a^	72.45^ab^	75.09^ab^	78.87^b^	2.68	p<0.05
Egg weight (g)	66.18	66.91	66.6	67.6	0.81	0.659
Egg mass (g/hen/day)	44.12^a^	48.46^ab^	49.97^bc^	53.27^c^	1.41	p<0.05
Feed conversion ratio	2.39^b^	2.17^a^	2.10^a^	2.04^a^	0.06	p<0.05
Mortality %	8.33	8.33	4.16	0	2.26	0.207

NC=No addition of GPA and OEO. GPA=50 ppm of bacitracin zinc. 80OEO=80 ppm of *Lippia origanoides* essential oil. 150OEO=150 ppm of *Lippia origanoides* essential oil. SEM=Standard error of the mean. Means with a common letter are not significantly different (p>0.05)

### Egg quality

In terms of the internal egg quality, there were no significant differences in the % of yolk, albumin, or shell among the treatments (p>0.05) (Table-3). However, the 150OEO treatment had a significantly greater albumin height and HU value than the NC and GPA groups (p<0.05), and both OEO treatment groups had significantly higher yolk color scores than the NC and GPA groups (p<0.05). In terms of the external egg quality, the shell thickness was significantly greater for the 150OEO and 80OEO treatment groups than for the NC and GPA groups (p<0.05), but the breaking force did not show any significant differences among the treatments (p>0.05) (Table-3). The OEO treatment groups also had significantly lower values of L and significantly higher values of a* and b* for the color of the shell than the GPA and NC groups (p<0.05).

**Table-3:** Effect of the inclusion of oregano essential oil on the egg quality parameters.

Variables	NC	GPA	80OEO	150OEO	SEM	p-value
Albumin height (mm)	7.15^a^	7.30^a^	7.50^a^	7.97^b^	0.13	p<0.05
Haugh units (HU)	82.69^a^	83.33^a^	84.02^a^	86.86^b^	0.80	p<0.05
Yolk color	10.18^a^	10.37^a^	10.9^b^	11.05^b^	0.09	p<0.05
% yolk	25.52	25.91	25.36	25.67	0.26	0.505
% albumin	64.45	63.99	64.53	64.6	0.35	0.610
% shell	10.03	10.1	10.11	10.14	0.10	0.879
Shell thickness (μm)	376.93^a^	387.65^ab^	388.75^b^	395.4^b^	0.39	p<0.05
Shell breaking force (N)	49.37	49.14	47.39	49.15	1.72	0.836
Shell color						
Luminosity (L)	65.16^c^	65.38^c^	63.41^a^	64.06^b^	0.21	p<0.05
Red (a*)	14.59^b^	14.26^a^	15.59d	15.08^c^	0.10	p<0.05
Yellow (b*)	26.37^a^	26.42^a^	26.99^b^	26.83^b^	0.10	p<0.05

NC=No addition of GPA and OEO. GPA=50 ppm of bacitracin zinc. 80OEO=80 ppm of *Lippia origanoides* essential oil. 150OEO=150 ppm of *Lippia origanoides* essential oil. SEM=Standard error of the mean. Means with a common letter are not significantly different (p>0.05)

### Intestinal morphometry

The morphometric analysis of the small intestine revealed significant variations in the height and width of the villi and the depth of the crypts in ISA Brown hens (Table-4). In the duodenum, the 150OEO, 80OEO, and GPA treatment groups had significantly higher villus heights than the NC group (p<0.05). In addition, the 150OEO treatment group had a significantly higher villus width and villus height:crypt depth ratio than the GPA and NC groups (p<0.05) and a significantly lower crypt depth than the GPA and 80OEO treatment groups (p<0.05). In the jejunum, the 150OEO, 80OEO, and GPA treatment groups had significantly higher villus heights and widths than the NC group (p<0.05). In addition, the 150OEO treatment group had a significantly lower crypt depth than the GPA and 80OEO treatment groups and a significantly higher villus height:crypt depth ratio than all other groups (p<0.05). In the ileum, the villus height, villus width, and villus height:crypt depth ratio of the 150OEO, 80OEO, and GPA treatment groups were significantly different from those of the NC group (p<0.05), but no significant differences were observed in the depth of the crypts (p>0.05).

**Table-4:** Effect of the inclusion of oregano essential oil on the morphometry of the small intestine in Isa Brown hens.

Parameters	NC	GPA	80OEO	150OEO	SEM	p-value
Duodenum						
Villus height (μm)	906.1^a^	1254.2^b^	1271.6^b^	1309.7^b^	7.77	p<0.05
Villus width (μm)	107.0^a^	105.9^a^	115.0^ab^	123.6^b^	4.47	p<0.05
Crypt depth (μm)	119.5^a^	145.9^b^	140.4^b^	120.4^a^	5.05	p<0.05
Ratio VH/CD	8.1^a^	8.8^a^	9.2^a^	11.0^b^	0.38	p<0.05
Jejunum						
Villus height (μm)	725.0^a^	1179.9^b^	1183.8^b^	1239.1^b^	9.08	p<0.05
Villus width (μm)	86.1^a^	103.5^b^	103.3^b^	107.4^b^	3.08	p<0.05
Crypt depth (μm)	105.9^ab^	112.6^b^	111.4^b^	100.7^a^	2.96	p<0.05
Ratio VH/CD	6.9^a^	10.5^b^	10.7^b^	12.5^c^	0.29	p<0.05
Ileum						
Villus height (μm)	542.9^a^	631.5^b^	650.0^b^	666.7^b^	9.82	p<0.05
Villus width (μm)	90.7^a^	115.8^b^	112.5^b^	113.3^b^	4.14	p<0.05
Crypt depth (μm)	150.2	146.5	145.4	142.5	5.34	0.786
Ratio VH/CD	3.7^b^	4.4^a^	4.5^a^	4.9^a^	0.21	p<0.05

NC=No addition of GPA and OEO. GPA=50 ppm of bacitracin zinc. 80OEO=80 ppm of *Lippia origanoides* essential oil. 150OEO=150 ppm of *Lippia origanoides* essential oil. SEM=Standard error of the mean. VH/CD=Villus height/crypt depth ratio. Means with a common letter are not significantly different (p>0.05)

## Discussion

The results obtained for laying performance in the present study agree with those reported by Ortiz *et al*. [21] who found that the inclusion of essential oil extracted from *L. origanoides* Kunth. in diets enriched with polyunsaturated fatty acids did not affect the average egg weight. Similarly, Özek *et al*. [22] showed that the addition of a mixture of essential oils based on herbs, organic acids, or mixed additives to a corn and soybean diet did not significantly affect egg weight but did tend to increase egg production compared with the control diet. However, He *et al*. [13] demonstrated that the addition of 100 mg/kg OEO to the feed significantly increased the average egg weight and the feed conversion ratio compared with the negative control and the antibiotic control (10% colistin sulfate) in Hy-Line hens. This variation in the effects of OEO treatment on productive parameters among studies can be attributed to differences in the genetic lines of birds used, the contents of carvacrol and thymol, the subspecies of plant from which the OEO was extracted, the administration methods, and the doses used.

There was no significant difference in the mortality of the birds among the treatments. However, the only treatment that gave 0% mortality was 150OEO. The low mortality of birds and increased egg production in the 150OEO treatment group are due to the addition of OEO affecting the eubiosis of the microbiota of the gastrointestinal tract, which is directly related to uterine health [23], and favoring the establishment of beneficial microorganisms, which improves the digestibility of nutrients, the immune response, and the resistance to pathogens in birds [24], optimizing the extraction efficiency and energy and nutrient use by birds [13,25,26].

Thymol and carvacrol exert antimicrobial effects on the structure of the bacterial cell wall by denaturing and coagulating its proteins, disrupting its structure, and increasing its permeability to hydrogen and potassium ions, which disrupts vital cell processes such as electron transport, protein translocation, phosphorylation, and other enzyme-dependent reactions; this then results in a loss of chemosmotic control of the cells, leading to bacterial death [27]. Consequently, these compounds can help improve intestinal health, which is reflected in improved performance and lower mortality.

The egg quality results partially agree with those of Abdel-Wareth [28] who found that Hy-Line Brown hens that were fed a diet supplemented with thymol (250 mg/kg), synbiotic (250 mg/kg), or a combination of the two (250 mg/kg each) had significantly higher HU values than the control group that was fed a basal diet but no significant difference in the percentage of yolk. However, Ding *et al*. [29] indicated that supplementation of the diet of laying hens with 50, 100, or 150 mg/kg of the commercial product Enviva EO, which contains 13.5% thymol and 4.5% cinnamaldehyde, did not significantly affect the albumin height or HU value. Similarly, He *et al*. [13] concluded that the addition of 50, 100, or 150 mg/kg OEO to the diet of Hy-Line hens did not significantly affect the HU value compared with the negative control and antibiotic control (10% colistin sulfate) groups; however, the 50 mg/kg diet did significantly increase the percentage of yolk compared with the antibiotic control group, though there was still no difference from the negative control. An increase in the HU value is important for the poultry industry, as it is related to increased protein quality and egg freshness [30]. The use of phytogenic additives with antibacterial and antioxidant properties, such as OEO, can improve the quality of the albumin, as previously reported by Bozkurt *et al*. [31] who added a blend of phytogenic essential oils, including OEO, to the diets of 52- to 61-week-old laying hens. Moreover, the bioactive ingredients in herbal plants have been shown to protect the magnum and uterus and to stimulate albumin secretion during laying [32].

The yolk color score was significantly higher when the diet was supplemented with OEO than in the NC and GPA groups. This is considered to be due to the ability of the main components of essential oils and organic acids such as thymol, sorbic acid, and fumaric acid to maintain fat-soluble constituents such as carotenoids and Vitamin D, which contribute to the color of the yolk [33]. These results agree with those of Wang *et al*. [33] who observed that 30-week-old hens that were fed a diet supplemented with 150 or 450 ppm of essential oils and organic acids had a higher yolk color than hens that were fed the control diet. By contrast, Ding *et al*. [29] did not observe differences in yolk color between hens that were fed a diet supplemented with the commercial product Enviva EO and those that were fed the control diet.

The results for the external quality of the egg obtained in the present study agree with those of Ding *et al*. [29] who observed that supplementation with the commercial product Enviva EO significantly increased the thickness of the eggshell but did not affect the resistance of the shell compared with the control group. Similarly, Karadağoğlu *et al*. [34] observed a significant increase in the thickness of the eggshell in laying hens that were administered 0.1, 0.2, or 0.3 mL/L of essential oil based on oregano, and Abdel-Wareth [28] found that the thickness of the eggshell increased in layers that were supplemented with 250 mg/kg thymol. However, the results of the present study contrast with those of Wang *et al*. [33] who found that supplementation with 450 or 300 ppm of essential oils and organic acids (200 g/kg sorbic acid, 200 g/kg fumaric acid, and 100 g/kg thymol) did not affect the thickness of the shell but did significantly increase its breaking strength compared with the control, while Cufadar [35] observed that the shell percentage, breaking force, and thickness were not affected by the addition of different levels of OEO. The increase in the thickness of the shell in treatments supplemented with OEO with a high content of thymol could be related to its effect on the metabolic activity of bacteria in the gastrointestinal tract of layers, which would positively influence the rate of absorption of minerals, particularly Ca^2+^ and Mg^2+^ [36]. Regarding the color of the shell, the OEO treatments resulted in lower values of L, indicating a darker shell surface [37]. Thus, supplementation with OEO gave a more intense shell coloration that tended toward brown, which is an important quality aspect in terms of consumer perception [38].

Hens that were supplemented with OEO exhibited morphological differences in their intestines, with increased width and height of the villi compared with the NC group. This supports the findings of Peric *et al*. [39] who stated that the gastrointestinal tract can adapt and react morphologically to external factors related to changes in diet. Thymol can have a positive impact on intestinal morphology by affecting the secretion of endogenous digestive enzymes [40]. This effect is very important because the main function of the villi is the absorption of nutrients, and this activity partially depends on their size, with the absorption ability increasing with an increased villus length [41] and crypt depth [13]. Deeper crypts indicate that faster cell turnover is occurring to allow villus renewal in response to normal shedding or inflammation induced by pathogens or their toxins [42]. The results of the present study agree with those of He *et al*. [13] who observed a significant increase in the villus height and the villus height:crypt depth ratio in the duodenum of hens supplemented with 100 mg/kg OEO compared with the negative control group. Similarly, Reis *et al*. [43] found that the height of the intestinal villi and the depth of the crypts were higher in broilers treated with 0.5% or 1.0% of a phytogenic feed additive (carvacrol, thymol, and cinnamaldehyde). By contrast, while Wang *et al*. [33] observed a significant increase in the height of the villi in the ileum of hens supplemented with 300 ppm of essential oils and organic acids, they did not observe any such differences in the duodenum or jejunum or in the depth of the crypts or the villus height:crypt depth ratio of the intestinal regions. However, Mousavi *et al*. [44] showed that supplementation with 100 or 200 ppm of a mixture of essential oils that contained thymol as one of the active ingredients resulted in an increase in the villus height and the villus height:crypt depth ratio in the jejunum of layers. These authors considered that the increased villus height:crypt depth ratio may have been due to an increase in the height of the villi as a result of the bioactive compounds that were present in the essential oils, as these components can induce changes in the histology of the gastrointestinal tract, the chemical composition of the digestive juices, the secretion of digestive enzymes, and the tissues related to the gastrointestinal tract, and thus can improve the digestibility of the diet leading to better growth and performance.

## Conclusion

Essential oil extracted from *L. origanoides* improves egg production and quality and also has a positive impact on the intestinal morphometry of ISA Brown laying hens. These findings demonstrate that this oil can be used as an alternative to GPAs in the production of laying hens, helping to address the global issue of increasing antibiotic resistance. However, further studies with laying hens are recommended.

## Authors’ Contributions

SYR and LMP designed and executed the experiment. SYR, LMP, and MAO collected the data and performed the experimental work. SYR, LMP, and MAO analyzed the data and drafted the manuscript. All authors read and approved the final manuscript.
